# Surrogacy of one-year survival for overall survival in advanced hepatocellular carcinoma

**DOI:** 10.1186/s12885-024-12000-7

**Published:** 2024-02-23

**Authors:** Yuzhi Jin, Hui Ren, Qianhua Yue, Wei Wu, Chuan Liu, Yixuan Guo, Peng Zhao

**Affiliations:** 1 Department of Medical Oncology, The First Affiliated Hospital, School of Medicine, Zhejiang University & National Key Laboratory of Advanced Drug Delivery and Release Systems, Zhejiang University, Hangzhou, 310058 China; 2 Sichuan Academy of Chinese Medicine Sciences, Chengdu, 610000 China; 3 The First Affiliated Hospital, Zhejiang University School of Medicine & Key Laboratory of Cancer Prevention and Intervention, Ministry of Education, 79 Qingchun Road, Hangzhou, China

**Keywords:** Hepatocellular carcinoma, Surrogate endpoint, Milestone survival, Overall survival

## Abstract

**Background:**

The increasing number of sequential treatments complicates the evaluation of overall survival (OS) in clinical trials for hepatocellular carcinoma (HCC), therefore, reliable surrogate endpoints (SEs) are required. This study aimed to evaluate the surrogacy of progression-free survival (PFS) and one-year (1-yr) milestone survival for OS in HCC trials.

**Methods:**

We systematically searched databases for randomized clinical trials that evaluated systemic treatments for advanced HCC. Individual patient data were reconstructed to calculate the 1-yr survival rate. We adopted a two-stage meta-analytic validation model to evaluate the correlation between SEs and OS, and the correlation between treatment effects on SEs and OS. The hazard ratio (HR) was calculated to assess the treatment effects on PFS and OS, and the 1-yr survival ratio was calculated to evaluate the treatment effects on the 1-yr milestone survival.

**Results:**

Thirty-two HCC trials involving 13,808 patients were included. A weak correlation was detected between the median PFS and median OS (R2 = 0.32), whereas the correlation improved between PFS HR and OS HR (R2 = 0.58). We identified strong correlations between the 1-yr survival rate and median OS and between the 1-yr survival ratio and OS HR (R2 = 0.74 and 0.65, respectively). In subgroup analyses, PFS HR strongly correlated with OS HR in trials relevant to immune checkpoint inhibitors (ICIs). Although the correlation remained weak between PFS and OS even in trials with PFS HR ≤ 0.6, the 1-yr survival rate and 1-yr survival ratio were strong surrogates for median OS and OS HR, respectively (R2 = 0.77 and 0.75).

**Conclusions:**

One-year milestone survival outperformed PFS as a SE for OS in HCC, indicating the application of 1-yr survival as a secondary endpoint. In particular, PFS HR was a potential SE for OS HR in the ICI trials.

## Background

Liver cancer is the sixth most common malignancy worldwide, with hepatocellular carcinoma (HCC) accounting for 75% of the cases [1]. Despite improved surveillance in high-risk populations, more than 60% of HCCs have become advanced at the time of diagnosis [2]. Survival remains poor in patients with advanced HCC for whom systemic therapy is the pivotal treatment strategy [3]. For decades, clinical trials have driven the development of systemic drugs for advanced HCC as first- and second-line treatments [4–9]. Among the endpoints for clinical research, overall survival (OS) is the most robust and valuable; however, it requires a long time to obtain and may delay clinical access to effective treatments [10]. Additionally, the advent of subsequent treatments complicates OS [10]. In this context, surrogate endpoints (SE) are becoming increasingly important for assessing the treatment effect more objectively and in a timely manner.

An ideal SE should predict the clinical endpoint early and accurately [11]. Progression-free survival (PFS), time-to-progression (TTP), and objective response rate (ORR) are common SEs for OS; however, their consistency with OS is only moderate in HCC clinical trials [12–14]. The evaluation of these SEs is mainly based on radiological changes, which are vulnerable to interpretation biases [10]. Furthermore, the Response Evaluation Criteria in Solid Tumors (RECIST) was initially proposed based on experience with cytotoxic agents; however, the mechanisms of targeted drugs and immune checkpoint inhibitors (ICIs), two common types of systemic drugs for HCC, are markedly different from those of traditional chemotherapy [15]. Although modified RECIST (mRECIST), which incorporates the assessment of viable tumors, was developed to compensate for this deficiency, few studies have evaluated whether mRECIST can improve the performance of common SEs for OS in HCC trials [16].

Several studies have reported that milestone survival is a potential SE for OS; however, it has barely been explored as a systemic treatment for HCC. For instance, one-year (1-yr) milestone survival outperformed PFS in terms of surrogacy for OS in lung cancer trials [17, 18]. Compared with radiology-based endpoints, milestone survival can be assessed more objectively and simply, and can also capture events related to the deterioration of liver function and general condition.

In this study, we aimed to evaluate the surrogacy of PFS and ORR for OS in HCC trials by exploring the efficiency of systemic treatments and to investigate whether 1-yr milestone survival could be a reliable SE for OS by reconstructing individual survival data from HCC trials.

## Methods

### Selection of eligible clinical trials

Comprehensive research was performed using PubMed, EMBASE, and the Cochrane Central Register of Controlled Trials. Both MeSH and free-text words were used to identify potentially eligible studies. We retrieved studies published between July 2008 (publication time of the SHARP trial for sorafenib) and March 2022. This restriction on publication time could reduce the heterogeneity due to the lack of standard treatment. All randomized trials investigating systemic treatments for HCC were potentially eligible. The exclusion criteria were as follows: 1) absence of Kaplan–Meier curves for PFS or OS; 2) single-arm trials; 3) non-randomized control; 4) only locoregional treatments involved in either the experimental or control arm; 5) systemic drugs for neoadjuvant or adjuvant treatments; 6) trials involving dose escalation; 7) post hoc or subgroup analyses of trials; 8) no median survival time in either the experimental or control arm; and 9) survival curves unsuitable for extracting data. For eligible clinical trials, relevant publications were searched and reviewed to obtain the latest survival data. Two investigators independently reviewed the studies for eligibility, and discrepancies were discussed by all the authors to reach a consensus.

### Data extraction and reconstruction of individual patient data

For eligible trials, the following data were independently extracted by two investigators: bibliographic information, systemic drugs, study design, sample size of each arm, ORR, median PFS, TTP, and OS. Hazard ratios (HRs) and corresponding 95% confidence intervals (95% CI) were also extracted to assess the treatment effects. For studies without reported HRs, we calculated PFS HR, TTP HR, and OS HR from the median survival time according to the method described by Tierney et al. [19].

To reconstruct individual patient data (IPD) for evaluating the 1-yr survival rate, we used DigitizeIt software V2.2 (https://www.digitizeit.xyz/) to extract IPD from PFS or TTP and OS Kaplan–Meier curves. Simultaneously, we extracted the number of patients at risk and outcome events. The Guyot algorithm was then adopted to assemble patients with predicted events of interest and survival times [20]. The Cox proportional hazards model was used to evaluate the HR for PFS and HR for OS of the reconstructed IPD.

### Statistical analysis

The surrogacy of SEs for OS was assessed using a meta-analytic two-stage validation model, which requires that the two conditions be met simultaneously for valid surrogacy [21]. Condition 1 required that SEs strongly correlate with OS, implying that patients achieving better SEs tend to live longer. Condition 2 requires a strong association between the treatment effects of SEs and OS, indicating that the treatment effect on SEs can reliably predict treatment effects on OS.

We evaluated the associations between SEs and OS using weighted linear regression (WLR) analysis, which can calculate the coefficient of determination (R^2^) at the trial level. The 95% CI of R^2^ was estimated using bootstrapping with 1000 replicates. The surrogacy level was assessed by the degree of correlation, which was quantitatively reflected by the R^2^ [22]. According to the criteria by Bernard et al., an *R*^2^ > 0.6 was defined as an indication for clinical relevance [23]. For studies not reporting PFS data, TTP was adopted as an alternative to PFS since the Pearson correlation (R) between these two endpoints can reach to 0.99 in HCC clinical trials [10]. As for the treatment effects on the ORR and 1-yr survival rate, the ratio was calculated between the experimental and control arms. A linear relationship test (F-test) was performed before the WLR analysis to verify the linear relationship between the two variables. Subgroup analyses were conducted based on the following classifications: 1) whether ICIs were involved; 2) whether locoregional treatments (LT) were involved; 3) trial phases; 4) treatment settings; 5) publication years; and 6) the value of HR for PFS.

*P* values were considered statistically significant at a two-sided *P*-value of < 0.05. All statistical analyses were performed using R software version 3.5.0 (R Program for Statistical Computing).

## Results

### Characteristics of the eligible clinical trials

**Fig. 1:**
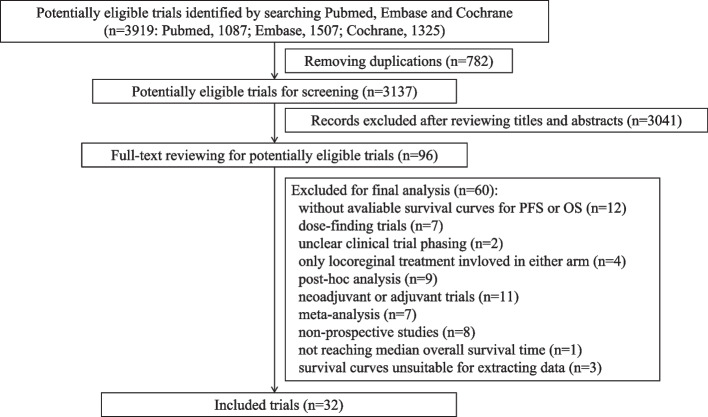
The flowchart of trial selection

After the initial research, we identified 3919 articles, of which 782 were excluded because of duplication. After reviewing the titles and abstracts, 96 articles were evaluated by reading the full texts, and 32 eligible trials were included in this study. The detailed selection process is illustrated in Fig. 1.

The baseline characteristics of 32 trials are summarized in Table 1, and a total of 13,808 patients were enrolled.

**Table 1:** Characteristics of eligible clinical trials included in the study

Number	Trial^a^	Year of publication	Treatment setting	Trial phase	Arms	Sample size	ORR by RECIST	Median PFS (months)	Median OS (months)	One-year survival rate (%)
1	NCT00108953	2010	1st	II	Doxorubicin Plus Sorafenib	47	0.04	6.0	13.7	50.47
					Doxorubicin Plus Placebo	49	0.02	2.7	6.5	28.57
2	CELESTIAL / NCT01908426	2018	2nd or 3rd	III	Cabozantinib	470	0.04	5.2	10.2	44.71
					placebo	237	0.004	1.9	8.0	31.56
3	NCT01507168	2016	≥ 2nd	II	codrituzumab	125	0.008	2.6	8.7	35.56
					placebo	60	0	1.5	10	28.19
4	NCT01287585	2018	≥ 2nd	III	ADI-PEG 20	424	0.008	2.6	7.8	31.70
					placebo	211	0.028	2.6	7.4	35.54
5	NCT01015833	2019	1^st^	III	Doxorubicin Plus Sorafenib	180	0.1	4.0	9.3	43.59
					Sorafenib	176	0.054	3.7	9.4	37.14
6	Shukui Qin	2021	1^st^	II-III	Donafenib	328	0.046	3.7	12.1	51.26
					Sorafenib	331	0.027	3.6	10.3	47.18
7	RESORCE / NCT01774344	2017	2^nd^	III	Regorafenib	379	0.07	3.4	10.6	40.86
					placebo	194	0.03	1.5	7.8	28.44
8	NCT01009593	2014	1^st^	III	Linifanib	514	0.101	5.4	9.1	48.91
					Sorafenib	521	0.061	4.0	9.8	41.04
9	KEYNOTE-240 / NCT02702401	2019	2^nd^	III	Pembrolizumab	278	0.183	3.8	13.9	57.86
					placebo	135	0.044	2.8	10.6	46.94
10	NCT00492752	2009	1^st^	III	Sorafenib	150	0.033	2.8	6.5	21.98
					placebo	76	0.013	1.4	4.2	11.29
11	NCT00699374	2013	1^st^	III	Sunitinib	530	0.066	3.6	7.9	35.92
					Sorafenib	544	0.061	3.0	10.2	43.80
12	IMbrave150 / NCT03434379	2020	1^st^	III	Atezolizumab + Bevacizumab	336	0.298	6.9	19.2	60.69
					Sorafenib	165	0.114	4.3	13.4	52.79
13	Ann-Lii Cheng	2016	1^st^	II	Dovitinib	82	0.06	4.11	8.07	28.73
					Sorafenib	83	0.101	4.18	8.56	42.98
14	REACH-2 / NCT02435433	2019	2^nd^	III	Ramucirumab	197	0.05	2.8	8.5	38.79
					Placebo	95	0.01	1.6	7.3	28.15
15	REACH / NCT01140347	2015	2^nd^	III	Ramucirumab	283	0.07	2.8	9.2	40.57
					Placebo	282	0.0007	2.1	7.6	34.97
16	NCT02774187	2019	1^st^	III	Sorafenib + HAIC	125	0.408	7.03	13.37	52.64
					Sorafenib	122	0.025	2.6	7.13	17.99
17	UMIN000005703	2016	1^st^	II	Sorafenib + HAIC	65	0.217	3.1	10.6	41.12
					Sorafenib	41	0.073	2.8	8.7	33.33
18	NCT01075555	2019	1^st^	III	Sorafenib + pravastatin	162	0.079	5.0	10.7	42.61
					Sorafenib	161	0.112	4.4	10.5	48.96
19	NCT01210495	2015	2^nd^	II	Axitinib + BSC	134	0.097	3.6	12.7	52.71
					Placebo + BSC	68	0.029	1.9	9.7	37.48
20	REFLECT / NCT01761266	2018	1^st^	III	Lenvatinib	478	0.188	7.4	13.6	53.61
					Sorafenib	476	0.065	3.7	12.3	45.84
21	S-CUBE / JapicCTI-090920	2017	2^nd^	III	S-1	222	0.05	2.6	11.1	45.19
					placebo	111	0.009	1.4	11.2	44.35
22	NCT02029157	2020	2^nd^ or 3^rd^	III	Tivantinib	134	0.052	2.8	10.3	39.63
					placebo	61	0.098	2.3	8.5	32.32
23	NCT01214343	2018	1^st^	III	Sorafenib + HAIC	102	0.36	4.8	11.8	48.10
					Sorafenib	103	0.18	3.5	11.5	44.92
24	NCT02329860	2021	≥ 2^nd^	III	Apatinib	261	0.11	4.5	8.7	35.98
					Placebo	132	0.02	1.9	6.8	33.54
25	NCT00825955	2013	2^nd^	III	Brivanib	263	0.12	4.2	9.4	38.88
					Placebo	132	0.02	2.7	8.2	32.69
26	NCT00105443	2008	1^st^	III	Sorafenib	299	0.02	5.5	10.7	41.39
					Placebo	303	0.01	2.8	7.9	30.30
27	NCT01829035	2019	1^st^	III	Sorafenib + cTACE	170	0.118	5.2	12.8	53.21
					Sorafenib	169	0.059	3.6	10.8	44.62
28	NCT01755767	2018	2^nd^	III	Tivantinib	226	0	2.1	8.4	38.29
					Placebo	114	0	2.0	9.1	38.83
29	NCT02528643	2021	≥ 2^nd^	II	Enzalutamide	110	0.018	2.23	7.75	28.45
					Placebo	55	0	1.87	7.69	31.48
30	NCT03009461	2022	1^st^	II	Sorafenib + HAIC	32	0.41	9.0	16.3	37.78
					Sorafenib	32	0.03	2.5	6.5	14.29
31	CheckMate459 / NCT02576509	2022	1^st^	III	Nivolumab	371	0.15	3.7	16.4	52.57
					Sorafenib	372	0.07	3.8	14.7	54.89
32	NCT0901901	2015	1^st^	III	Sorafenib + Erlotinib	362	0.066	3.2	9.5	39.12
					Sorafenib + Placebo	358	0.039	4.0	8.5	40.65

^a^Each trial can be identified with clinical trial registration number. For the trials without referring number, the first authors of the corresponding papers were listed

Among these studies, three investigated ICIs, and four trials referred to LTs. Twenty-four trials were in phase III, seven studies were in phase II, and the remaining one was in phases II–III. Eighteen trials were in the first-line setting and the rest were in secondary or later-line settings. In terms of publication years, nine out of 32 trials were published between 2008 and 2015, and the rest were published between 2016 and 2022. Regarding treatment efficiency, 18 trials showed a significant difference in PFS, whereas only 13 trials provided significant survival benefits in OS.

### Overall analysis of different SEs for OS

**Fig. 2:**
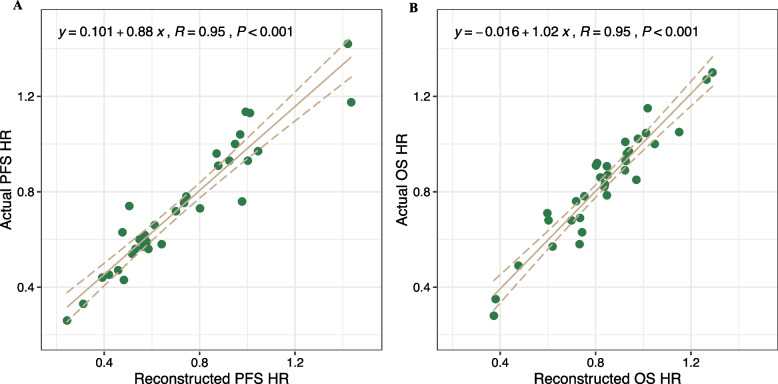
The validation of agreement between the reconstructed and original individual patient data (IPD). Progression free survival (PFS) (**A**) and overall survival (OS) (**B**) hazard ratios calculated from reconstructed IPD had good consistency with those obtained from original IPD

Before evaluating the surrogacy of the 1-yr survival for OS, we assessed the agreement between the reconstructed and original IPD. As shown in Fig. 2, the PFS HR and OS HR calculated using the reconstructed IPD were consistent with those obtained using the original IPD.

**Fig. 3:**
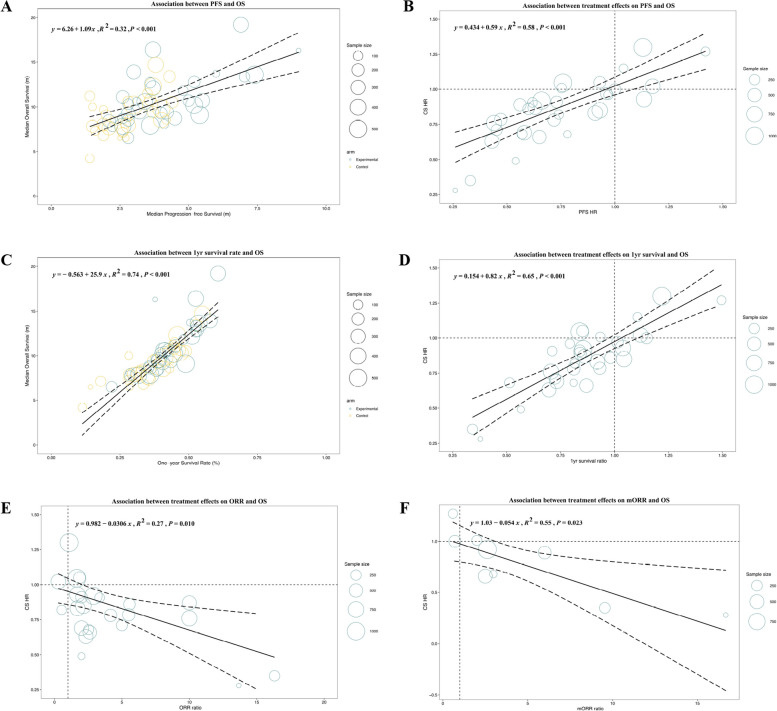
Performance of different surrogate endpoints for overall survival (OS). The size of the circle represents sample size. The correlations were weak between median progression-free survival (PFS) and median OS (**A**), and between PFS hazard ratio (HR) and OS HR (**B**). One-year (1-yr) milestone survival strongly correlated with OS: 1-yr survival rate—median OS (**C**) and 1-yr survival ratio—OS HR (**D**). There was a weak correlation between objective response rate evaluated by RECIST v1.1 (ORR) ratio and OS HR (**E**), while the correlation was stronger between ORR evaluated by mRECIST (mORR) ratio and OS HR (**F**)

Based on data from 32 trials, a weak correlation was observed between the median PFS and median OS (R^2^ = 0.32, 95% CI: 0.08–0.55) (Fig. 3A). In terms of treatment effects, there was a moderate correlation between PFS HR and OS HR (R^2^ = 0.58, 95% CI: 0.40–0.79) (Fig. 3B). A strong correlation was detected between the 1-yr survival rate and the median OS (R^2^ = 0.74, 95% CI: 0.63–0.88) (Fig. 3C). As shown in Fig. 3D, the consistency between the 1-yr survival ratio and OS HR was higher than that of PFS HR (R^2^ = 0.65, 95% CI: 0.47–0.99). As shown in Fig. 3E, the ORR had a weak correlation with the OS HR (R^2^ = 0.27, 95% CI: 0.03–0.56). The ORR ratio is the ratio of the ORR in experimental arms to the ORR in control arms, which is intended to reflect the treatment effect of the target regimen. The correlation between the mORR ratio and OS HR (R2 = 0.55, 95% CI: 0.07–0.90) was stronger (Fig. 3F).

### Subgroup analyses of different SEs for OS

We performed subgroup analyses stratified by treatment type, trial phase, treatment setting, publication year, and PFS HR value and yielded similar findings (Table 2).

**Table 2:** Detailed results of surrogacy assessment on different endpoints

	Subgroups	Included trials	Included patients	R2	95% confidence interval
ORR ratio – OS HR	All trials	24	11,924	0.27	0.03–0.56
mORR ratio – OS HR	All trials	9	2,960	0.55	0.07–0.90
PFS					
Median PFS—median OS	All trials	32	13,808	0.32	0.08–0.55
	ICIs relevant	3	1,657	0.70	0.01–1.00
	ICIs irrelevant	29	12,151	0.41	0.16–0.67
	LT relevant	4	714	0.80	0.56–0.98
	LT irrelevant	28	13,094	0.31	0.08–0.56
	Phase II	7	983	0.44	0.09–0.86
	Phase III	25	12,825	0.31	0.05–0.54
	First-line setting	18	8,415	0.30	0.04–0.64
	secondary or later-line setting	14	5,393	0.13	0.02–0.42
	2008–2015	9	4,915	0.21	0.02–0.62
	2016–2022	23	8,893	0.39	0.13–0.65
	PFS HR ≤ 0.6	11	3,928	0.48	0.16–0.80
	PFS HR > 0.6	21	9,880	0.30	0.03–0.63
PFS HR – OS HR	All trials	32	13,808	0.58	0.40–0.79
	ICIs relevant	3	1,657	0.86	0.00–1.00
	ICIs irrelevant	29	12,151	0.61	0.42–0.82
	LT relevant	4	714	0.68	0.15–1.00
	LT irrelevant	28	13,094	0.58	0.39–0.82
	Phase II	7	983	0.78	0.38–0.99
	Phase III	25	12,825	0.55	0.33–0.81
	First-line setting	18	8,415	0.57	0.29–0.96
	secondary or later-line setting	14	5,393	0.73	0.45–0.89
	2008–2015	9	4,915	0.54	0.08–0.95
	2016–2022	23	8,893	0.62	0.45–0.82
	PFS HR ≤ 0.6	11	3,928	0.40	0.01–0.82
	PFS HR > 0.6	21	9,880	0.37	0.05–0.70
1-yr survival					
1-yr survival rate – median OS	All trials	32	13,808	0.74	0.63–0.88
	ICIs relevant	3	1,657	0.51	0.02–0.99
	ICIs irrelevant	29	12,151	0.70	0.57–0.88
	LT relevant	4	714	0.46	0.01–0.99
	LT irrelevant	28	13,094	0.75	0.64–0.88
	Phase II	7	983	0.55	0.19–0.98
	Phase III	25	12,825	0.76	0.05–0.89
	First-line setting	18	8,415	0.69	0.55–0.88
	secondary or later-line setting	14	5,393	0.84	0.64–0.94
	2008–2015	9	4,915	0.65	0.39–0.96
	2016–2022	23	8,893	0.77	0.68–0.86
	PFS HR ≤ 0.6	11	3,928	0.77	0.61–0.98
	PFS HR > 0.6	21	9,880	0.75	0.62–0.92
1-yr survival ratio – OS HR	All trials	32	13,808	0.65	0.47–0.99
	ICIs relevant	3	1,657	0.49	0.00–1.00
	ICIs irrelevant	29	12,151	0.72	0.41–0.87
	LT relevant	4	714	0.90	0.57–1.00
	LT irrelevant	28	13,094	0.64	0.44–0.98
	Phase II	7	983	0.76	0.45–1.00
	Phase III	25	12,825	0.63	0.19–0.84
	First-line setting	18	8,415	0.64	0.24–0.87
	secondary or later-line setting	14	5,393	0.66	0.20–0.86
	2008–2015	9	4,915	0.74	0.12–0.95
	2016–2022	23	8,893	0.66	0.33–0.86
	PFS HR ≤ 0.6	11	3,928	0.75	0.42–0.95
	PFS HR > 0.6	21	9,880	0.42	0.12–0.84

*Abbreviations*: *ORR* Objective response rate, *OS* Overall survival, *HR* Hazard ratio, *mORR* ORR Evaluated by mRECIST, *PFS* Progression-free survival, *ICIs* Immune checkpoint inhibitors, *LT* Locoregional treatment

**Fig. 4:**
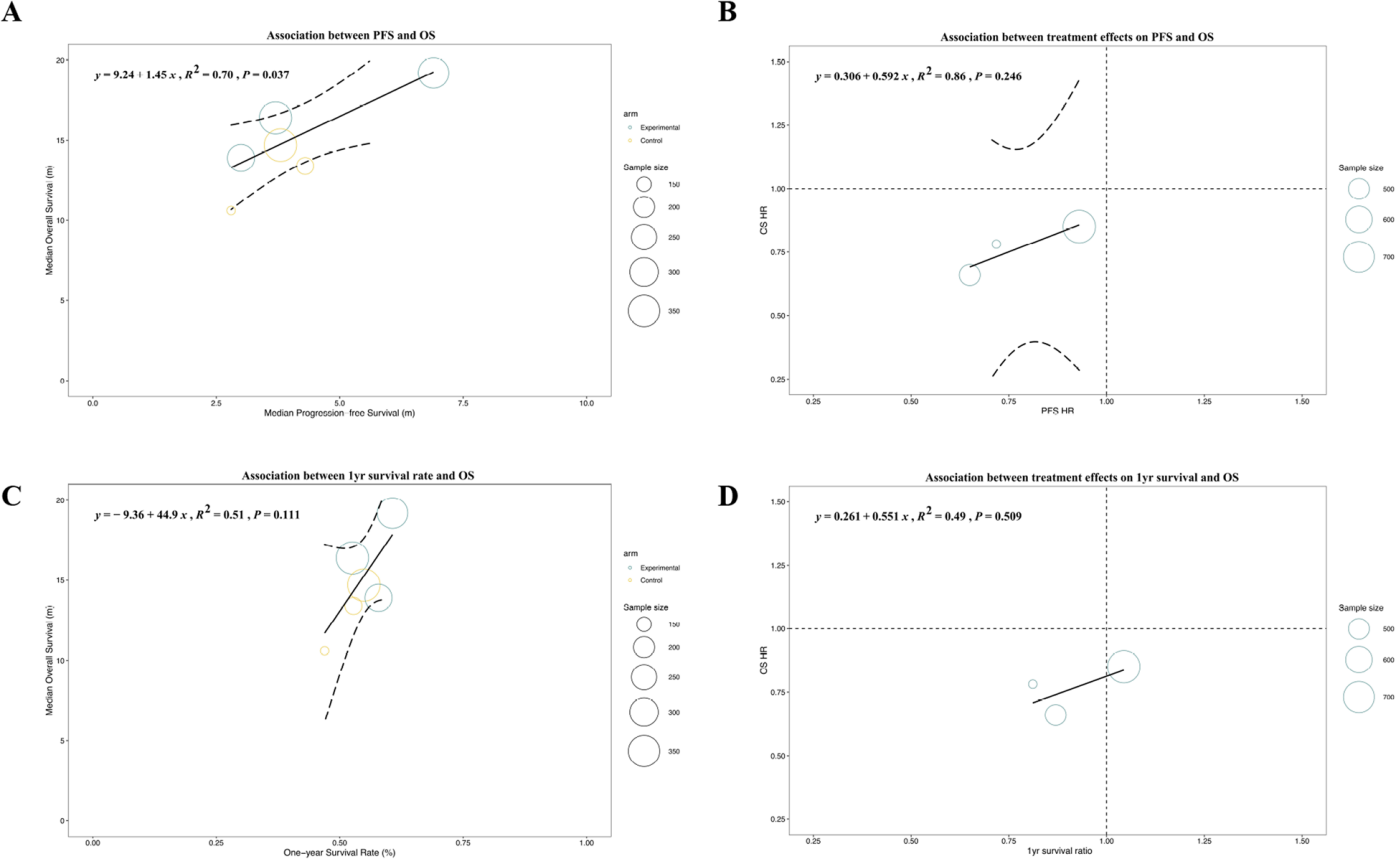
Subgroup analyses in trials relevant to immune checkpoint inhibitors (ICIs). The correlation was strong between progression-free survival (PFS) and overall survival (OS) (**A**, **B**). One-year milestone survival did not strongly correlate with OS in ICI trials (**C**, **D**)

In the analyses of subgroups with ICIs involvement, the correlation between median PFS and median OS was strong (Fig. 4A: *R*^2^ = 0.70, 95% CI: 0.01–1.00), and there was an even better correlation between PFS HR and OS HR (Fig. 4B: *R*^2^ = 0.86, 95% CI: 0.00–1.00). Weaker correlation was observed between 1-yr survival rate and median OS in trials relevant to ICIs (Fig. 4C: R2 = 0.51, 95% CI: 0.02–0.99). The surrogate performance of 1-yr survival ratio for OS HR was worse than that for PFS HR in trials relevant to ICIs (Fig. 4D: *R*^2^ = 0.49, 95% CI: 0.00–1.00). So the 1-yr survival and PFS could be used as complementary surrogate endpoints for HCC patients receiving different treatment modalities.

**Fig. 5:**
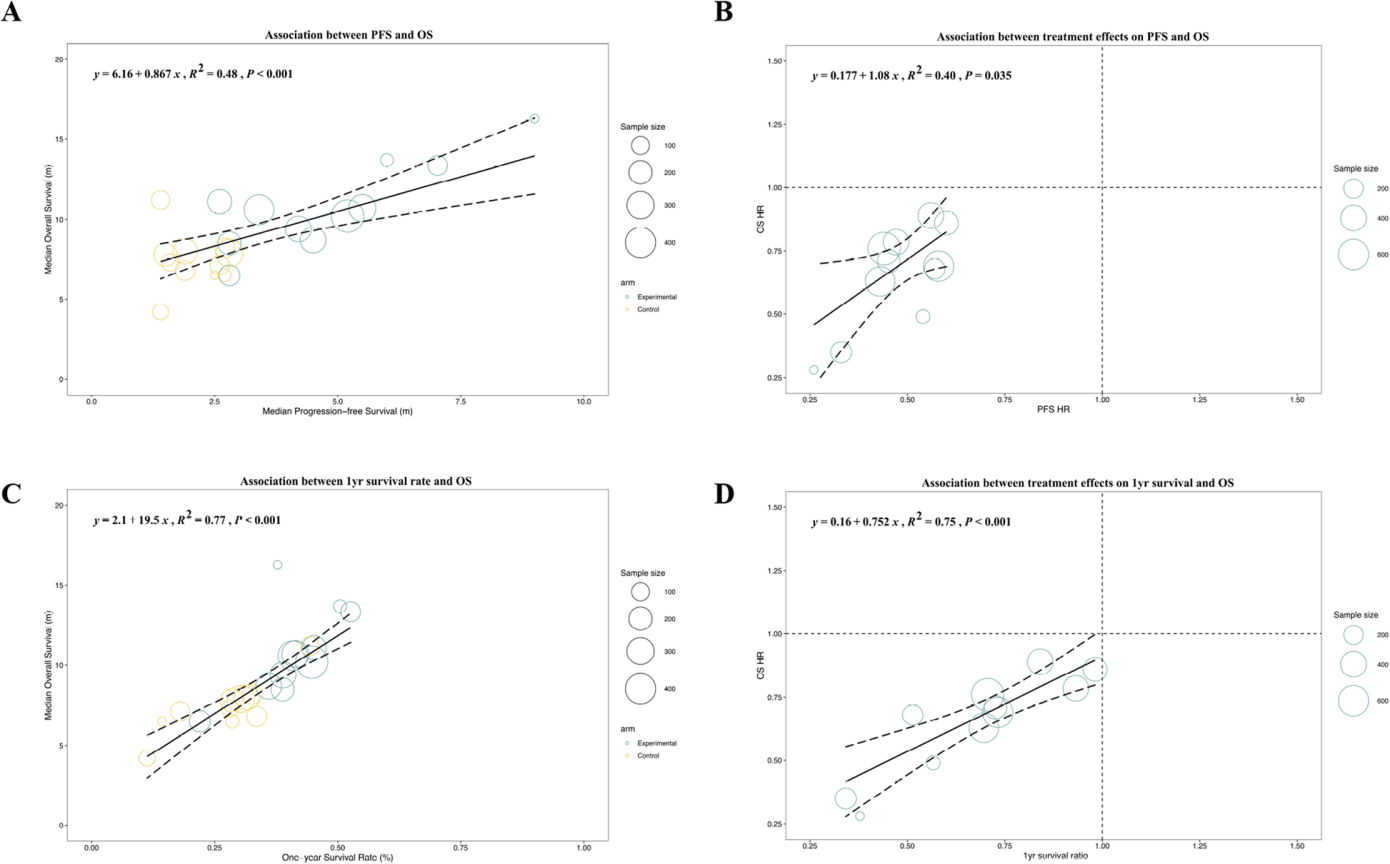
Subgroup analysis in trials with progression-free survival (PFS) hazard ratio ≤ 0.6. The correlation was insufficient between PFS and overall survival (OS) (**A**, **B**). One-year milestone survival strongly correlated with OS in HCC trials with PFS HR ≤ 0.6 (**C**, **D**)

In trials irrelevant to ICIs, the surrogacy of 1-yr survival was better than that of PFS, in terms of either absolute value or treatment effects. A similar tendency was found in both subgroups classified according to whether the LTs were referred to. Except for the strong association between the 1-yr survival rate and median OS in phase III trials, the surrogacy of SEs in phase II trials was better than that in phase III trials. The association between either the 1-yr survival ratio or PFS HR and OS HR was stronger in secondary- or later-line trials than in first-line trials. In recently published trials, the disassociation between 1-yr survival ratio and OS HR was more prominent, whereas an inverse relationship was observed in other SEs. We further classified the enrolled trials according to whether the PFS HR value was ≤ 0.6 [10]. In trials with PFS HR > 0.6, the association was weak for all SEs except 1-yr survival rate for median OS. For trials with PFS HR ≤ 0.6, PFS still showed weak correlations with OS (Fig. 5A and B: *R*^2^ = 0.48, 95% CI: 0.16–0.80; *R*^2^ = 0.40, 95% CI: 0.01–0.82). Conversely, 1-yr survival was strongly associated with OS in trials with PFS HR ≤ 0.6 (Fig. 5C and D: *R*^2^ = 0.77, 95% CI: 0.61–0.98; *R*^2^ = 0.75, 95% CI: 0.42–0.95).

## Discussion

To the best of our knowledge, this is the first study to assess the surrogacy of PFS and 1-yr milestone survival for OS in clinical trials of systemic treatment for advanced HCC. Using a two-stage meta-analytic validation model, we assessed the correlations between PFS or 1-yr survival and OS and the correlations between treatment effects evaluated by PFS or 1-yr survival and OS. We detected a strong correlation between 1-yr survival and OS, but a relatively weak correlation between PFS and OS. In subgroup analysis, there was a strong correlation between HR for PFS and HR for OS in trials relevant to ICIs. Although the correlation between PFS and OS remained weak even in trials with PFS HR ≤ 0.6, 1-yr survival was strongly correlated with OS in this subgroup, indicating that 1-yr survival was a potentially ideal complementary SE.

OS is an unquestionable and unbiased endpoint for assessing treatment efficiency in tumor-related clinical trials. However, reaching the OS endpoint in HCC is time-consuming, and the interpretation of OS can be confounded by post-progression treatments [24]. In this context, oncologists have evaluated the reasonability of indicators such as PFS and ORR as alternative endpoints for OS; however, their surrogacy is unsatisfactory in trials of advanced HCC [25]. Only 29% of HCC clinical trials met the primary endpoint, which is significantly lower than the success rate of 37% for other tumors [10]. A reliable SE could terminate ineffective treatments in a timely manner to protect the interests of the patients. Thus, this study aimed to explore an effective SE for OS in clinical trials of advanced HCC, based on a meta-analytic two-stage assessment model.

By analyzing 32 prospective and randomized clinical trials for advanced HCC, a weak correlation was detected between median PFS and median OS (*R*^2^ = 0.32), whereas the correlation was stronger between PFS HR and OS HR (*R*^2^ = 0.58). These results are comparable to previously reported findings. For instance, Cabibbo et al. reported a weak correlation between median PFS and median OS (*R*^2^ = 0.20), and they also reported that early PFS was a robust SE for early OS in trials of immunotherapy for HCC [12]. Unfortunately, they did not evaluate the correlation between HR for PFS and HR for OS, perhaps because of the inclusion of some single-arm trials. Based on these findings, it is essential to set up a control arm to calculate the HR value in phase II trials, as the median survival might not provide sufficient information for designing subsequent trials.

Furthermore, we detected a weak correlation between the ORR ratio and OS HR (*R*^2^ = 0.27), whereas a stronger correlation was observed between the mORR ratio and OS HR (*R*^2^ = 0.55). Sirisha et al. identified a weak correlation between the odds ratio (OR) of ORR and HR of OS (*R*^2^ = 0.13) [25]. Similarly, a disassociation also existed in HCC trials for systemic therapies between ORR OR and OS HR, and mORR OR outperformed ORR OR in terms of OS surrogacy [13]. The surrogate level of mORR for OS was also higher than that of ORR in our study. The RECIST criteria were initially proposed to assess the efficiency of cytotoxic drugs, which have different antitumor mechanisms from those of targeted drugs and ICIs. Although sorafenib provides clear survival benefits, the ORR is only 2% [8]. To overcome this poor correlation, mRECIST, which incorporates the concept of a viable tumor, was proposed to evaluate the response of patients with HCC receiving systemic treatment [16]. As the response assessed by mRECIST had a better correlation with OS than the response assessed by RECIST, it might be more appropriate to adopt mRECIST to evaluate the treatment response and disease progression in HCC clinical trials.

In this study, we identified strong correlations between 1-yr survival rate and median OS, and between 1-yr survival ratio and OS HR (*R*^2^ = 0.74 and 0.65, respectively). Milestone survival is a potential intermediate endpoint for capturing clinically meaningful activity [26, 27]. In a meta-analysis of trials for metastatic NSCLC, Blumenthal et al. found that 1-yr survival was strongly correlated with OS [17]. In addition, Shen et al. reported that 1-yr milestone survival had strong surrogacy for OS in previously treated advanced non-small cell lung cancer [18]. However, whether 1-yr milestone survival can predict OS in HCC clinical trials has not been elucidated. According to our results, 1-yr survival is a potentially valid SE for OS in trials of patients with advanced HCC. Unlike endpoints, such as PFS or ORR, the assessment of 1-yr survival is not based on imaging interpretation, which is relatively objective. Considering the good consistency between the 1-yr survival rate and median OS, 1-yr survival might be an ideal endpoint in single-arm phase II clinical trials. Although the survival curves for OS can be overlapped at the 1-yr time cutoff for some less malignant cancers, the survival curves separate clearly at this time for HCC patients due to the dismal prognosis.

In subgroup analyses, we found a strong correlation between HR for PFS and HR for OS in trials relevant to ICIs (*R*^2^ = 0.86), which might be due to the durable treatment efficiency of ICIs [28]. Although the sample size was limited for this subgroup, our results were similar to those of previous studies on lung cancer [18]. Given that PFS HR ≤ 0.6 is commonly recognized as a surrogate threshold for significant improvement in OS, we further classified the trials according to the PFS HR [10]. Weak correlations were detected between median PFS and median OS, and between PFS HR and OS HR in trials with either PFS HR ≤ 0.6 or PFS HR > 0.6. However, there were strong correlations between 1-yr survival rate and median OS, and between 1-yr survival ratio and OS HR in trials with PFS HR ≤ 0.6 (*R*^2^ = 0.77 and 0.75, respectively). These findings indicated that 1-yr survival is a potentially ideal SE for OS, which could complement the underperformance of PFS as a surrogate for OS. The performance of SEs can vary according to clinical context, patient characteristics, and study design. Although 1-yr milestone survival might not be the primary endpoint in HCC trials, it could become a complementary endpoint in assessing treatment efficiency in clinical trials for advanced HCC based on its strong correlation with OS detected in our study.

This study has several limitations. First, the number of trials was limited to evaluating the surrogacy of mORR and performance of SEs in trials relevant to ICIs. Although our results are consistent with previously reported findings, further validation using more prospective and randomized trials is warranted. Second, we used the reconstructed IPD to calculate 1-yr survival rates rather than the original IPD, which is not accessible. However, the reconstructed data exhibited excellent consistency with the original data. Third, we did not evaluate the surrogate performance of other endpoints for OS such as the duration of response and rates of adverse events.

## Conclusions

In conclusion, we identified strong correlations between 1-yr survival and OS in clinical trials for advanced HCC, indicating the application of 1-yr milestone survival as a surrogate endpoint for OS. Although PFS was weakly correlated with OS in HCC trials, PFS HR was strongly associated with OS HR in ICI trials, which could be a potential SE in HCC trials.

## Data Availability

The datasets generated and analyzed during the current study are available from the corresponding author upon reasonable request.

## Abbreviations

HCC: Hepatocellular carcinoma
OS: Overall survival
SE: Surrogate endpoint
PFS: Progression-free survival
TTP: Time-to-progression
ORR: Objective response rate
RECIST: Response Evaluation Criteria in Solid Tumors
ICI: Immune checkpoint inhibitor
mRECIST: Modified RECIST
1-yr: One-year
HR: Hazard ratio
CI: Confidence interval
IPD: Individual patient data
WLR: Weighted linear regression
LT: Locoregional treatment
OR: Odds ratio
